# Boron Removal from Aqueous Solutions by Using a Novel Alginate-Based Sorbent: Comparison with Al_2_O_3_ Particles

**DOI:** 10.3390/polym11091509

**Published:** 2019-09-16

**Authors:** Hary Demey, Jesus Barron-Zambrano, Takoua Mhadhbi, Hafida Miloudi, Zhen Yang, Montserrat Ruiz, Ana Maria Sastre

**Affiliations:** 1 Department of Chemical Engineering, Universitat Politècnica de Catalunya, ETSEIB, Diagonal 647, 08028 Barcelona, Spain; 2 Faculty of Chemical Engineering, Universidad Autonoma del Yucatan, Periférico Norte kilómetro 33.5, Tablaje Catastral 13615, Chuburna de Hidalgo Inn, Mérida, Yucatán, C.P. 97203, Mexico; 3 Laboratory of Environmental Biomonitoring (LBE), University of Carthage, Faculty of Sciences of Bizerte, Avenue de la République, 7021 Jarzouna, Tunisia; 4 Laboratory of Chemistry of Materials, University of Oran 1, B.P 1524 El M’naouer-Oran, Algeria; 5 School of Chemistry and Materials Science, Jiangsu Provincial Key Laboratory of Material Cycling and Pollution Control, Nanjing Normal University, Nanjing 210023, China

**Keywords:** alginate, alumina, boron, composite, novel sorbent, sorption

## Abstract

Boron removal was evaluated in the present work by using calcium alginate beads (CA) and a novel composite based on alginate–alumina (CAAl) as sorbents in a batch system. The effects of different parameters such as pH, temperature, contact time, and composition of alginate (at different concentrations of guluronic and mannuronic acids) on boron sorption were investigated. The results confirm that calcium alginate beads (CA) exhibited a better adsorption capacity in a slightly basic medium, and the composite alginate–alumina (CAAl) exhibited improved boron removal at neutral pH. Sorption isotherm studies were performed and the Langmuir isotherm model was found to fit the experimental data. The maximum sorption capacities were 4.5 mmol g^−1^ and 5.2 mmol g^−1^, using CA and CAAl, respectively. Thermodynamic parameters such as change in free energy (ΔG^0^), enthalpy (ΔH^0^), and entropy (ΔS^0^) were also determined. The pseudo-first-order and pseudo-second-order rate equations (PFORE and PSORE, respectively) were tested to fit the kinetic data; the experimental results can be better described with PSORE. The regeneration of the loaded sorbents was demonstrated by using dilute HCl solution (distilled water at pH 3) as eluent for metal recovery.

## 1. Introduction

Boron compounds are extensively used in the chemical industry for manufacturing of cosmetics, heat-resistant glasses, ceramics, and fire retardants materials. Boric acid is also widely applied as a disinfectant due to its antibacterial and antifungal properties [1]. World boron demand has increased over the last decade and is expected to increase over the following years; this fact has accelerated the introduction of this element into environmental ecosystems, and in the pollution of rivers and soils of European countries. Water containing boron may affect agricultural harvests, causing a negative impact on local economies. A typical example of this contamination, is the case of the Valencia region in Spain (where the densest area of ceramic industries in the country is located); wastewaters are treated with conventional methods before discharging into the public network, but specifically designed techniques for the removal of boron from the effluents are not available.

Although the growth of many plants needs trace amounts of boron, this element at high concentrations becomes harmful to both plants and animals [1]. According to the statements of the World Health Organization (WHO), clear influences on the male reproductive system after either short- or long-term exposure have been found: dogs, mice, and rats developed testicular lesions when fed with boric acid or borax. The upper limit of the concentration of boron in drinking water has been set to 2.4 mg∙L^−1^ by the WHO [2].

Boron could be toxic in harvests depending on the strength of the plants in resisting high levels of this element [3]. Valencia is the region in Spain characterized by the highest production of citric fruits (such as orange and lemon), and also is the focus of the major porcelain production of the country. However, these fruits are among the most sensitive to boron compounds. Important losses in agricultural fields have been reported by government authorities due to the high concentration of boron in waters. Innovative technologies must be developed to provide solutions to this environmental concern.

Besides boron, ceramic industries also use metals oxides during the manufacturing process of frits glaze (typically ZnO); zinc oxide is utilized as a fluxing agent in the preparation of frits and enamels, improving the elasticity by reducing the change in viscosity (as a function of temperature). Therefore, Zn(II) is largely present with boron in the ceramic effluents (frits production).

Several methods for boron and zinc removal from aqueous solutions have been previously reported. These methods include electrodialysis [4] precipitation [5], chemical coagulation and electrocoagulation [6,7], complexation/nanofiltration [8], phytoremediation [9], ion-exchange [10,11], reverse osmosis [12,13,14], and sorption [15,16,17,18,19,20]. Biopolymer-based materials could be an economical alternative technology for the removal of metalloids and heavy metals from aqueous solutions. The use of biopolymers for boron sorption has received little attention over the last decade. Published papers report the possibility of using chitosan modified with N-methyl-D-glucamine [21], cotton cellulose [22], and sugars [23]; the potential abilities of alginates for boron removal and its application as an encapsulating material have been demonstrated as well [24,25,26,27]. 

Alginate is a polysaccharide existing in brown algae, which are abundant in different marine ecosystems of the earth. Alginate consists of β-D-mannuronic and α-L-guluronic acids; these contain hydroxyl groups on their molecular backbones, making the biopolymer reactive towards boron compounds (esters formation). In turn, when divalent ions coexist, a three-dimensional hydrogel network could be formed, which typically follows the egg-box model [28].

The addition of functional groups into the biopolymer matrix may increase the reactivity and/or the strength of the resulting sorbent; low operating costs and the availability of the feedstock are the main criteria for the selection of competitive materials. Alumina is one of the most common sorbents used in recent years, since aluminum sources are present throughout the world; however, alternative sustainable materials have to be developed for reducing and recycling for the utilization of mineral compounds. Bouguerra et al. [29] developed sorption of boron using alumina particles (particle size <200 µm) and the results were compared with the conventional reverse osmosis systems. Although alumina is able to remove boron, Bouguerra et al. [29] did not study the elution step and it is not clear whether the particles can be reused. A good sorbent conceived for large-scale applications, needs to be recyclable and have ease of manipulation in order to be competitive.

The aim of the present work was to employ calcium alginate (CA)-based materials for boron removal from waters; the possibility of using a new alginate/alumina-based composite is reported to facilitate the handling of alumina on sorption processes. Sorption capacities of commercial samples, namely alumina and CA were tested for comparison. The equilibrium, as well as kinetic, sorption data were evaluated in detail.

## 2. Materials and Methods

### 2.1. Materials 

Boron solutions were prepared by dissolving boric acid (H_3_BO_3_, provided by Merck KGaA, Darmstadt, Germany) in Milli-Q water. The alumina (Al_2_O_3_) particles, and the salts of sodium and calcium were supplied by Panreac (Barcelona, Spain). The content in mannuronic and guluronic acids (M and G blocks, respectively) was determined by an NMR spectroscopy technique. The molar ratio was estimated as 64% M-blocks and 36% G-blocks [25].

### 2.2. Preparation of Sorbents

The sorbent manufacturing can be summarized in three steps [25]: i) Two liters of sodium alginate solution (2% *w*/*w*) were obtained by dissolving 40 grams of sodium alginate in 1960 g of pure water; the mixture was mechanically stirred at 300 rpm for 4 h to achieve full homogenization. Then, the stirring speed was changed to 50 rpm for 2 h, to dissipate the air and to avoid bubble formation. Alginate solution was divided into two different recipients (of one liter) and these were labeled as A and B. ii) Twenty grams of alumina were carefully introduced into the B recipient and it was kept under agitation (at 300 rpm) overnight. iii) Four liters of ionotropic gelation solution of calcium nitrate (0.05 M) were prepared and divided into two recipients of two liters each one (C and D solutions).

Then, the A and B solutions were systematically dosed under continuous agitation into C and D solutions, respectively. The produced beads were identified as CA (calcium alginate) and CAAl (composite alginate–alumina). The scheme of the manufacturing process is represented in Figure S1 (in the Supplementary Materials Section). A volume of 500 mL of each sorbent material was air-dried at 20 °C for 72 h. The average diameter of the beads was determined by using a Malvern-Mastersizer 3000^TM^ instrument; it was 2.3 mm and 0.8 mm for hydrogel and air-dried beads, respectively.

### 2.3. Characterization of Sorbents

#### 2.3.1. Scanning Electron Microscopy

Surface morphology of the beads was analyzed using a JEOL JSM 7100F field emission scanning electron microscope (JEOL Ltd., Peabody, MA, USA), equipped with an Energy Dispersive X-ray (EDX) spectrometer (INCA 250, Oxford instruments, Oxford, UK). The sorbent samples were analyzed before and after sorption of boron from aqueous solutions through EDX-technique; although the detection of boron is difficult due to the low photon energy of this element (which emits low energy peaks close to the electronic noise of the detection system), the main elements of the natural alginates were identified.

#### 2.3.2. Thermal-Gravimetric Analyses

Thermal-gravimetric analyses were performed under N_2_ atmosphere (with a flow rate of 50 mL min^−1^) at a heating rate of 10 °C min^−1^ using a Perkin-Elmer TGA 6 instrument (Waltham, MA, USA). The samples of 5 mg were degraded within a temperature range of 30–300 °C.

### 2.4. Batch Sorption Experiments

The pH values were adjusted to be within the range of 3 to 12 by using 0.1 M NaOH and/or 0.1 M HCl aqueous solution (as required). An amount of 0.7 g of sorbent was added to 100 mL of boron solution (50 mg L^−1^) at room temperature (20 ± 1 °C). After 72 h of agitation (at 150 rpm) using an orbital shaker (Rotabit J.P. Selecta, Barcelona, Spain), the solutions were forced through 1.2 µm filtration membranes and the filtrates were analyzed using a microwave plasma-atomic emission spectrometer 4100 MP-AES equipment (from Agilent technologies, Melbourne, Australia) at a wavelength of 249.7 nm. The sorption capacity was calculated by means of Equation (1). 

Equilibrium experiments (sorption isotherms) were carried out by mixing 0.7 g of adsorbent with 100 mL of boron solutions at different concentrations (10–500 mg L^−1^) and agitated for 72 h. The initial pH of the boron solutions was adjusted to 11. The residual boron concentration was determined using 4100 MP-AES equipment.

The uptake kinetics experiments were performed by adding (under continuous stirring) a known amount of adsorbent (i.e., 1.75 g) to 250 mL of boron solution (50 mg L^−1^) at a fixed initial pH 11; aliquots of adsorbate were withdrawn for analysis at different times over 72 h of contact (at 150 rpm). The collected samples were then filtered through a 1.2 µm filtration membrane. The kinetic profiles were compared for two different configurations of the beads:

- Hydrogel beads (Ø 2.3 mm)
- Air-dried beads (Ø 0.8 mm)

The sorption uptake and the sorption efficiency of the materials were obtained through Equations (1) and (2):

(1) $$q=\frac{V\left(C_{0}-C_{\mathrm{eq}}\right)}{m}$$

(2) $$\%\;\mathrm{SE}=\frac{\left(C_{0}-C_{\mathrm{eq}}\right)}{C_{\mathrm{eq}}}\cdot 100$$

where q is the sorption capacity of sorbents (mg g^−1^), % SE is the boron sorption efficiency, C_0_ is the initial boron concentration (mg L^−1^), Ceq is the boron concentration at equilibrium (mg L^−1^), m is the mass of adsorbent (g), and V is the volume of the boron solution (L). All experiments were carried out at room temperature.

## 3. Fitting of Sorption Isotherms and Kinetics

### 3.1. Sorption Isotherms

Equilibrium data are commonly described by the Langmuir and Freundlich models (Equations (3) and (4), respectively). These models allow the distribution of the boron species between the adsorbent and the solution interface to be described [30]:

(3) $$q=\frac{q_{\max}{\mathrm{bC}}_{\mathrm{eq}}}{1+{\mathrm{bC}}_{\mathrm{eq}}}$$

(4) q=KFCeq1n

where q_max_ is the maximum adsorption capacity of sorbents (mg g^−1^), and C_eq_ is the equilibrium concentration in the solution. In the Langmuir model, *b* is related to the energy of adsorption (L mg^−1^). The parameters *n* and *K_F_* are Freundlich adsorption constants, indicative of adsorption intensity and relative capacity, respectively. 

### 3.2. Kinetic Studies

The effect of the contact time is typically described by using the pseudo-first-order (PFORE) [31] and pseudo-second-order (PSORE) rate equations [32,33]. 

PFORE:

(5) $$\frac{{\mathrm{dq}}_{t}}{d_{t}}=K_{1}\left(q_{1}-q_{t}\right)$$

Integrating for the boundary conditions *t* = 0 to *t* = *t* and *q*_t_ = 0 to *q*_t_ = *q*_t_:

(6) $$\log\left(q_{\mathrm{eq}}-q_{t}\right)=\log(q_{\mathrm{eq}})-\frac{K_{1}}{2.303}t$$

PSORE:

(7) $$\frac{{\mathrm{dq}}_{t}}{{\left(q_{\mathrm{eq}}-q_{t}\right)}^{2}}=K_{2}d_{t}$$

Integrating for the boundary conditions t = 0 to t = t and q_t_ = 0 to q_t_ = q_t_:

(8) $$\frac{1}{q_{t}}=\frac{1}{K_{2}q_{\mathrm{eq}}^{2}}+\frac{1}{q_{\mathrm{eq}}}t$$

where q_eq_ is the equilibrium sorption capacity (mg g^−1^), qt is the sorption capacity (mg g^−1^) at any time t (min), k_1_ and k_2_ are the pseudo-constants of the models (g mg^−1^ min^−1^). 

The intraparticle diffusion was obtained with Equation (9) (Weber and Morris equation, W&M) [34]:

(9) $$q_{t}=K_{p}t^{1/2}+C$$

where C is the intercept, and Kp is the intraparticle diffusion rate constant.

### 3.3. Effect of Temperature

Two temperatures (20 °C and 35 °C) were selected to assess the temperature effect on the removal performance using CA and CAAl as sorbents. Van’t Hoff Equation (10) was used to determinate the thermodynamic parameters of the sorption process: standard entropy (ΔS°), standard enthalpy change (ΔH°) and free energy change [19]:

(10) $${\text{lnK}}_{\text{C}}=-\frac{\Delta \text{H}{}^{\circ}}{\text{RT}}+\frac{\Delta \text{S}{}^{\circ}}{\text{R}}$$

Gibbs free energy changes (ΔG°) were determined according to ref. [19]:

(11) $$\Delta G{}^{\circ}=-{\mathrm{RTlnK}}_{C}$$

(12) $$K_{C}=\frac{C_{S}}{C_{\mathrm{eq}}}$$

where T is the solution temperature (K), Kc is the equilibrium constant, R is the gas constant (8.314 J mol^−1^ K^−1^), Cs is the equilibrium concentration of boron onto the sorbent (mmol L^−1^), and Ceq is the equilibrium boron concentration (mmol L^−1^).

### 3.4. Towards an Application with Ceramic Wastewater

In order to evaluate further real effluents from the ceramic industries in the column system (which will be the scope of a future work), the assessment of the CAAl sorbent was first performed with Zn(II) ions in a batch system. The material was evaluated with Zn(II), since it is one of the most representative metals used in the frits production, and it is commonly found in coexistence with boron species [35]. The maximum sorption capacity and kinetic profiles for zinc removal were determined.

## 4. Results and Discussion

### 4.1. Characterization of the Materials

#### 4.1.1. SEM–EDX Analyses

**Figure 1:**
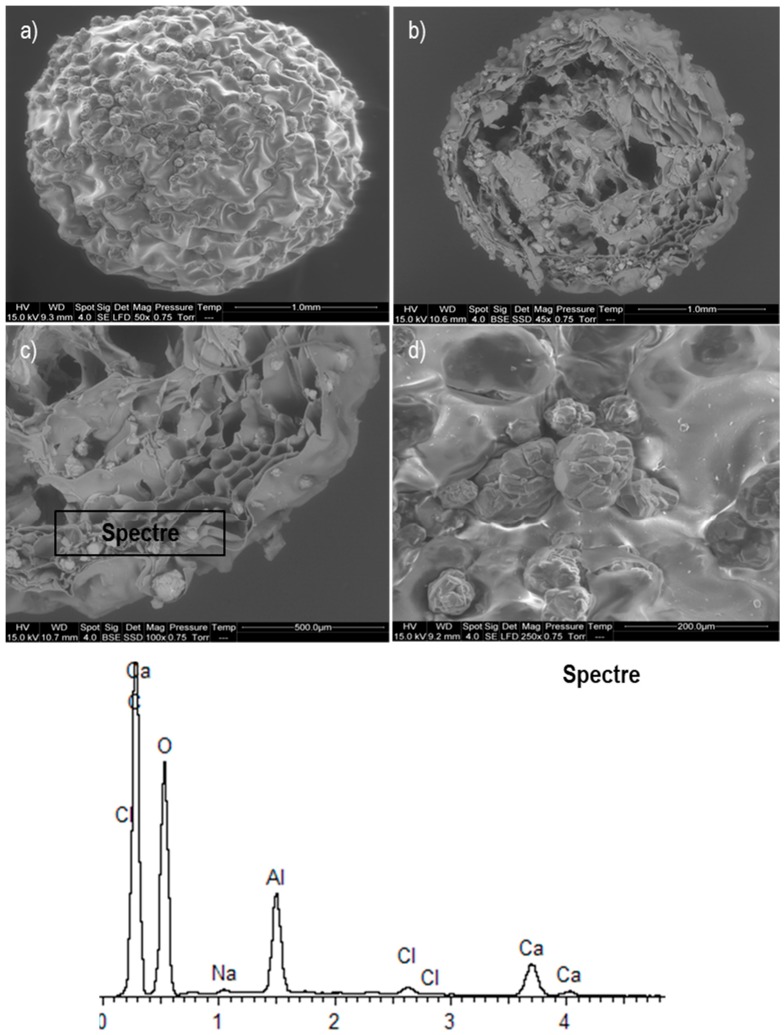
Scanning Electron Microscopy (SEM) images of the CAAl material: (**a**,**b**) Topography of the sorbent; (**c**) Energy Dispersive X-ray (EDX) analysis on the surface area of the sorbent, (**d**) Alumina particles immobilized in the alginate support.

The examination of the topography and surface structure of the sorbents was possible through SEM micrographs (Figure 1). The roughness of the material surface is reported in Figure 1a,b, the SEM images indicate the presence of some fissures that were formed during the drying process. Figure 1c shows the alumina particles which are attached to the calcium alginate network (onto the surface and in the whole volume of the beads).

Some beads were cross-sectioned to observe the distribution of alumina in the volume of the sorbent (Figure 1b). The alumina was not homogenously distributed into the beads and this may be due to the low agitation speed used during the manufacturing process; nevertheless, it was found that the alumina content in the composite was constant (2% *w*/*w* approximately). The spectrum in the cross-sectioned surface of the material (Figure 1c) demonstrates the presence of calcium, carbon, oxygen, alumina, chloride and sodium ions, as expected since these are the main elements of the composite. Some alumina particles can be observed embedded in the external area of the beads (Figure 1d); their presence could modify the surface charge of the sorbent, enhancing the removal of different metal ions [29].

#### 4.1.2. TGA

Thermal-gravimetric analyses of CA and CAAl were performed to examine the degradation of the materials with temperature. The TGA and derivative curves are presented in Figures S2a,b (in the Supplementary Materials Section). The thermal decomposition of polysaccharides include three different phases: (i) dehydration, (ii) depolymerisation (which is an adjunct to the rupture of C–O and C–C bonds in the ring units, as a result, the production of CO, CO_2_, and H_2_O molecules is increased), (iii) the development of poly-nuclear aromatic and graphitic carbon structures [36]. Both materials have similar degradation profiles with an initial weight loss (4% for CA, and 7% for CAAl) due to the residual moistures of the samples. Thermograms show that the decomposition of the adsorbents begins at 195.4 °C for CA, with a residual mass of 33% and at 210.2 °C for CAAl with a residual mass of 57%. These results show that these sorbents could even be used at high temperatures with CAAl having a better thermal stability; this being of scientific interest for further applications with CAAl material. 

### 4.2. Effect of pH

**Figure 2:**
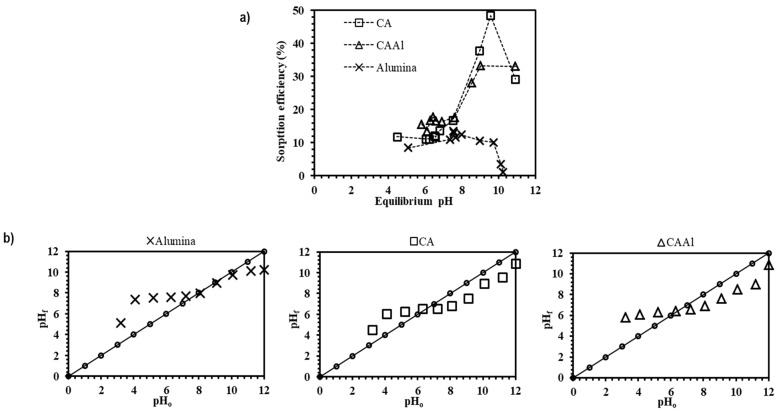
Influence of pH on boron removal. (**a**) Removal performances using alumina, CA and CAAl. materials (**b**) variation in pH (pH_0_: 3–12, [B]initial = 50 mg L^−1^, V = 100 mL, T room, 1 atm).

The effect of pH is a key factor on the sorption process. The pH may affect the speciation of the metals through formation of complex molecules [37], and the protonation of carboxylic groups present in the alginate chains (the mannuronic and guluronic blocks; i.e., M and G-blocks) [38]. The experiments were carried out over a pH range from 3 to 12; the results in Figure 2a demonstrated that higher sorption efficiency was obtained on decreasing the proton concentration of the solutions. Initially (over pH 3–9), the best sorption efficiency was obtained with CAAl material, which is the result of the individual contribution of alumina and alginate in this interval. However, from pH > 9 the best boron uptake is obtained with CA material (the sorption with alumina decreases with an increase in the pH).

In our previous studies, it was suggested that the binding reaction of boron species [B(OH)_3_ and B(OH)_4_^−^] occurs with the OH- groups in the cis-position on the reagent sorbent [26]. Thus, boron sorption onto CA, CAAl, and alumina is the result of the ester formation between the OH^−^ groups of the boron species and those present in the sorbent material. The removal of boron by alumina (pH 3–9) is attributed to both chemical and physical processes: i) interaction with hydroxyl ions, and ii) interaction with electrostatic forces between the sorbent and boron species. The results obtained with alumina agree with the trend presented by Seki et al. [16] and Bouguerra et al. [29], who reported a negative influence on boron removal with increasing pH (using alumina and alumina-based materials as sorbents).

The pH and concentration determine the chemical species of boron present in the solution. As the pKa for boric acid is 9.2 and the point of zero charge (pH_PZC_) for different types of alumina is around 8.7–9.0 [29], the surface of alumina is negatively charged at pH > pH_PZC_. Thus, anionic boron species [B(OH)_4_^−^] would have lower interactions with alumina (and derivatives materials), giving a lower boron uptake at pH >9; hence alginate present in the composite becomes the active material for boron sorption (Figure 2a). 

B(HO)_3_ + H_2_O ↔ B(HO)_4_^−^ + H^+^  pKa = 9.2 (13)

**Scheme 1:**
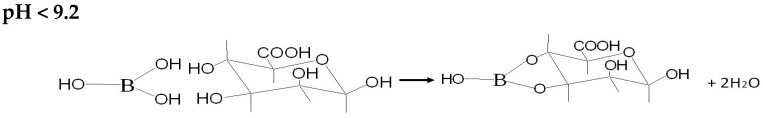
Interaction of boric acid specie with hydroxyl groups of calcium alginate.

**Scheme 2:**
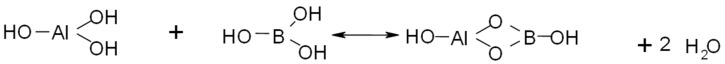
Interaction of boric acid specie with hydroxyl groups of alumina.

**Scheme 3:**
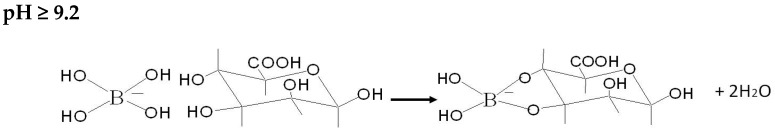
Interaction of tetra-hydroxy borate ions specie with hydroxyl groups of calcium alginate.

**Scheme 4:**
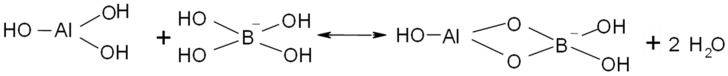
Interaction of tetra-hydroxy borate ions specie with hydroxyl groups of alumina.

Figure 2b presents the impact of the variation of boron solutions pH on the sorption process. Between pH 3 and 5, carboxylic groups in the alginate formed a weak base that neutralized the acid solution; this was verified by comparing the initial and final pH of boron solutions. Haug [39] reported pKa values of COO- groups in mannuronic and guluronic acids as 3.38 and 3.65 respectively; thus, at pH below 3.5 the carboxylic groups are protonated and the sorption of boron is negligible. At pH > 3.5 the protons of the solution compete with boric acid for the active sites and a “buffering effect” is produced. Upon increasing the pH, the proton concentration is lower and the sorption becomes more efficient (a similar trend was also reported by Fiol et al. [27]). The maximum removal of boron is obtained at equilibrium pH 9.5–9.8. The interaction of boron species with alginate and alumina can be summarized by the following Scheme 1, Scheme 2, Scheme 3 and Scheme 4:

It was previously reported that this mechanism takes place when the distances between OH- groups in the sorbent are similar to those for boric acid and borate ions [40,41].

### 4.3. Equilibrium Studies

**Figure 3:**
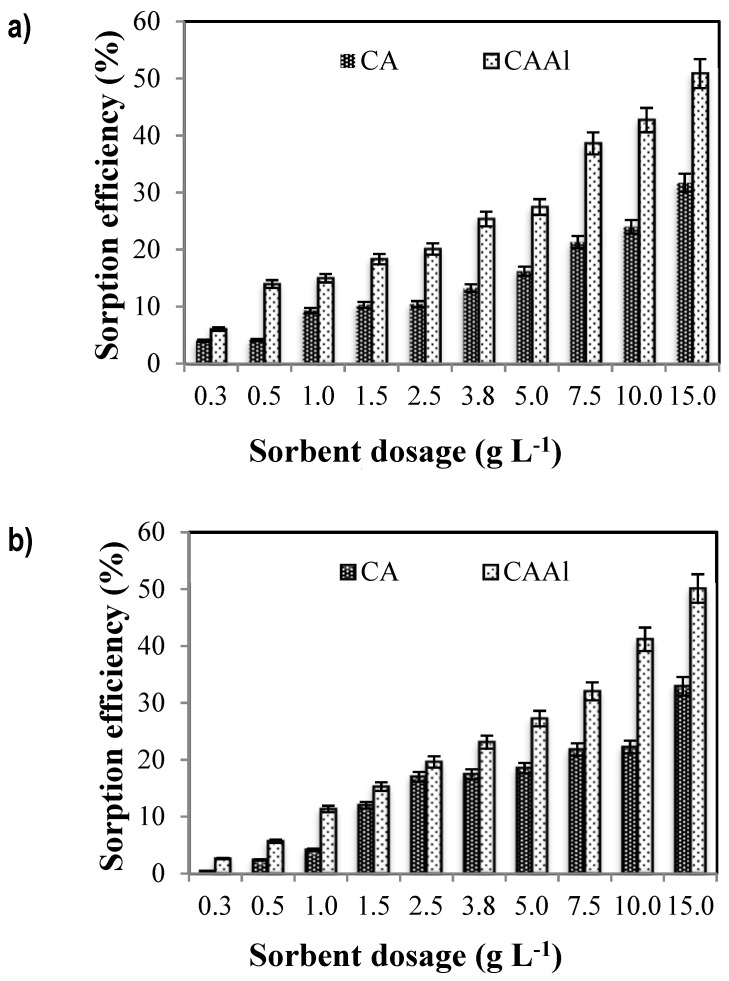
Effect of sorbent dosage on boron removal. (**a**) Removal performances using an initial boron concentration of 5 mg L^−1^ (**b**) Removal performances using an initial boron concentration of 25 mg L^−1^ (pH_0_: 7, equilibrium pH 6.6, [B]initial = 5 mg·L^−1^ and 25 mg·L^−1^, V = 100 mL, T room, 1 atm).

The effect of the sorbent dosage on boron removal was performed in order to select the best conditions for the process. Experiments were carried out at two different boron concentrations: 5 mg L^−1^ and 25 mg L^−1^ (Figure 3a,b, respectively). These low concentrations were chosen to ensure the presence of monomeric species of boron. According to Morisada et al. [3], at boron concentrations below 270 mg L^−1^ boric acid acts as a weak and monobasic acid; thus, monoborate ions become the dominant species in the solution (Figure S3 displays the boron and aluminum species involved in the process). In both cases, CAAl beads exhibited better boron uptake than CA, achieving about 30–40% boron adsorption when 7.5 g L^−1^ of sorbent were dosed, and 50% when 15 g L^−1^ were in contact with the polluted solution. These results could be due to the increase of sorption sites available for the process. Removal efficiency did not change significantly above a sorbent dosage of 7.5 g L^−1^.

**Figure 4:**
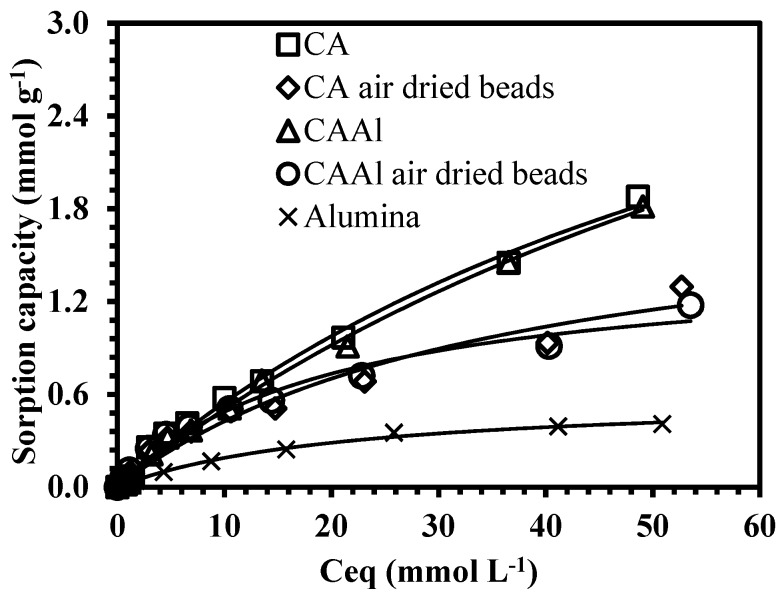
Equilibrium studies. (**a**) Boron sorption isotherms using hydrogel and air-dried beads. (**b**) Sorption isotherms using different types of alginates (pH_0_: 11, equilibrium pH: 9.5–9.8, V = 100 mL, T room, 1 atm).

Figure 4 plots the isotherm curves of boron: the isotherms are critical to characterize the optimal conditions of the process, and these allow the distribution of the sorbate between the liquid and solid phases at equilibrium to be described [42]. The curves indicate the appearance of a typical saturation plateau at high boron concentration. The presence of an initial steep slope is an indication of the high affinity between the sorbent and the sorbate molecules. The materials have a higher performance at low boron concentrations; these can remove more than 50% of initial boron content (at concentrations <50 mg L^−1^). Experimental data were fitted with the Langmuir equation with better accuracy than the Freundlich model (by direct comparison of the determination coefficients, Table 1). Langmuir was originally designed for systems in which the sorbent surface is occupied by a monolayer of sorbate molecules, nevertheless, the mathematical fitting of the experimental results does not necessarily mean that the hypotheses of the model are verified, but it is useful for interpreting the boron uptake. The maximum sorption capacities obtained were 4.5 mmol g^−1^ and 5.2 mmol g^−1^, using hydrogel beads of CA and CAAl, respectively.

Figure 4 also compares CA material (the standard hydrogel beads used in this work) with air-dried beads of calcium alginate. Isotherm plots exhibit an initial similar trend; nevertheless the results in Table 1 indicate that the drying procedure may influence the equilibrium uptake (the maximum sorption capacity estimated by the Langmuir model was found to be two times greater for hydrogel beads than air-dried beads). Probably, with the air-drying method the accessibility to all the active sites of the sorbents is reduced. It is in agreement with our previous work [43], in which we used algal-based material for heavy metal removal: two different drying configurations were tested: i) air-dried beads, and ii) freeze-dried beads. It was concluded that the uncontrolled air-drying technique may affect the diffusion process of the metal molecules into the sorbent. The beads after air-drying were found to have been shrunken; in turn, the freeze-dried sorbent conserved their spherical shape and particle size (and the sorption properties were similar to the original hydrogel beads). This useful information is necessary for the design of industrial systems, since the kinetic data could also be impacted.

**Figure 5:**
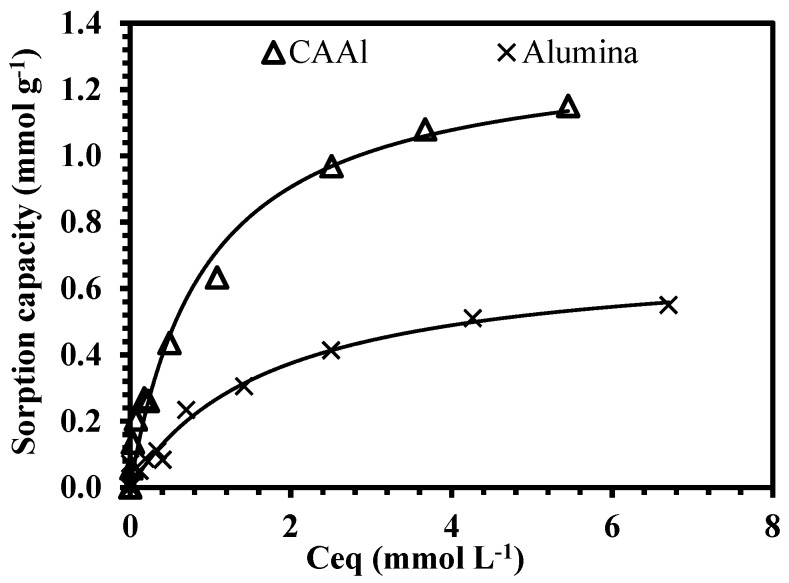
Equilibrium studies for zinc removal using alumina and CAAl materials as sorbents (pH_0_: 4, equilibrium pH: 5.4, Sorbent Dosage: 1 g L^−1^, T room, 1 atm).

Although the sorption capacities towards boron are similar for both sorbents (CA and CAAl), the introduction of alumina is important for improving the mechanical strength of the beads, enhancing the stability of the material. The presence of alumina also allows a better removal of several coexisting ions, usually present in the industrial ceramic effluents (such as zinc). Figure 5 shows the comparison of the CAAl material and alumina powder for the sorption of Zn(II), the immobilization of alumina into the alginate matrix results in a sorbent which can be used for different industrial pollutants. According to the Table 2, the Zn(II) sorption capacity increases from 0.7 mmol L^−1^ (using alumina as sorbent) to 1.3 mmol L^−1^ (using CAAl material); these preliminary tests are of great interest for the efficient treatment of real wastewaters in column systems, which will be the scope of a future work. 

Table 3 summarizes the comparison of the sorption capacities reported in the literature for several sorbents; boron removal by calcium alginate (CA) and composite alginate–alumina (CAAl) are in the same order of magnitude as the published materials. It is noteworthy that the performance of the commercial resin (AMBERLITE IRA-743), usually used for boron binding in waste waters, is considerably lower than the studied sorbents.

Furthermore, in order to study the influence of the uronic acids concentration (M and G blocks) on boron removal, the sorption isotherms onto CA (prepared from three different alginates) were performed (Figure S4). Two additional alginates, with a different molar composition to that used for manufacturing the standard beads of this work, were tested and compared with the commercial resin AMBERLITE IRA-743 (provided by Merck KGaA, Darmstadt, Germany); the technical characteristics provided by the fabricant of the resin are represented in Table S1 (Supplementary Materials Section). The calcium alginate beads (CA) were prepared with the alginates LF-200S and LF-240D (supplied by Acros Organics^TM^, a Thermo-Fischer scientific brand, Illkirch, France), the same procedure described in Section 2.2 was followed. The mannuronic and guluronic acid composition was previously reported by Demey et al. [43] and Bertagnolli et al. [54]:

- LF-200S: 63% G-blocks; 37% M-blocks
- LF-240D: 30% G-blocks; 70% M-blocks

The results indicate that boron uptake is independent of the percentage of M or G monomers present in alginates, It seems that the reactive hydroxyl groups of both monomers are equally available for boron sorption. The results have been compared with the commercial AMBERLITE-IRA 743 resin at the same operating conditions (Figure S3). As expected, the sorption capacity of CA beads is 5–6 times higher than the commercial resin, it indicates that alginate is an interesting support for manufacturing sorbents materials since: i) the raw biomass has low-cost; ii) it has excellent sorption properties, and iii) it is a renewable resource present in several marine ecosystems (brown algae). 

### 4.4. Sorption Kinetics

**Figure 6:**
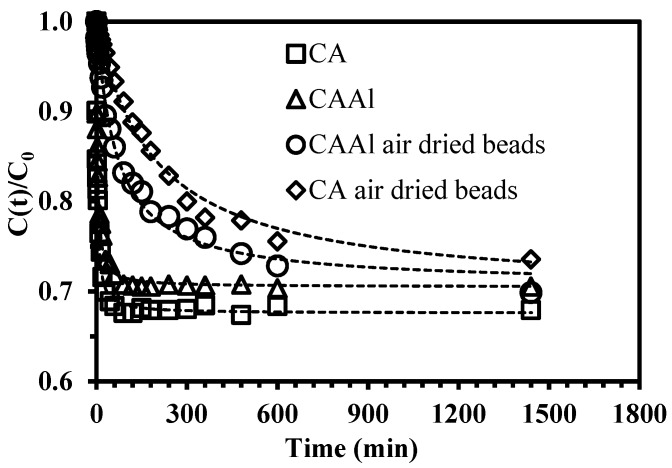
Boron sorption kinetics using hydrogel and air-dried beads (pH_0_: 11, equilibrium pH: 9.5–9.8, [B]initial = 50 mg·L^−1^, 1.75 g of sorbent, V = 250 mL, T room, 1 atm).

Kinetic studies were performed in order to evaluate the effect of contact time on boron sorption. Figure 6 plots the kinetic profiles of boron removal for both materials (CA and CAAl). The kinetics curves are characterized by an initial rapid sorption followed by a slow-rate step when approaching equilibrium.

The kinetic profiles for both hydrogel and air-dried beads are initially different: the first step of the hydrogel beads is faster that of the air-dried. In hydrogel beads the sorption sites (hydroxyl groups) are more easily available; as a consequence less contact time is required to attain equilibrium. The second step in the kinetic profiles is similar for both kinds of beads. The air-drying method has a strong impact on the required contact time for achieving the equilibrium (30 min was enough for the hydrogel beads and 200 min for air-dried beads to attain the saturation plateau).

The theoretical q values obtained from the pseudo-first-order and pseudo-second-order models are shown in Table 4. Both models fitted the experimental data well, the theoretical q values agree well with the experimental q_exp_ values. The simplified equation of Weber and Morris (W&M) for evaluating the contribution of the resistance to intraparticle diffusion was also tested (Equation (9)). The linear plot did not pass through the origin, verifying that intraparticle diffusion is not the unique step that controls the boron uptake kinetics (Figure S5). 

The resistance to film diffusion may affect the control of uptake kinetics. As expected, the Kp values obtained from the slope of the linear portions of the kinetics profiles (Table 4) are three times higher for hydrogel beads than those for air-dried sorbents. Thus, the uncontrolled air-drying technique makes the accessibility of the ions into the active sites difficult, which impacts on the metal diffusion and on the time for achieving the equilibrium. The results in Table 4 demonstrate that a better diffusion is carried out using the original hydrogel beads; this is not surprising, since the sorbents after air-drying are contracted and the spherical shape becomes non-regular for all beads (the spherical shape is deformed, Figure S6). 

Additionally, the mechanical strength of the sorbents was verified and monitored during the kinetic studies; CA beads tend to slightly swell (after 24 h of agitation time) due to the presence of sodium ions in the solution (NaOH was used for adjusting the initial pH to 11), which affects the stability of the “eggs-box” structure of calcium alginate. On the contrary, the CAAl material presented a better stability during the same interval of time, the average diameter (Ø) was found to be 3.0 mm for CA, and 2.5 mm for CAAl (which is similar to the original beads before sorption: Ø 2.3 mm).

### 4.5. Effect of Temperature

Table 5 reports the effect of temperature on boron removal using CA and CAAl as sorbents. The equilibrium constant (Kc) was found to decrease with increasing temperature of the solution; which means that the boron uptake is not favored at 308 K (the equilibrium constant decreases 6–7 times with an increase of the temperature from 293 K to 308 K). These results highlight the exothermic nature of the sorption process, which is confirmed by the negative values of the enthalpy change.

Kavak [19] and Polowczyk et al. [55] reported that a change in enthalpy (ΔH°) of 0 to 20 kJ mol^−1^ implies a physisorption, while a change between 80 to 400 kJ mol^−1^ (absolute values) corresponds to a chemisorption process. In this work, the enthalpy changes of CA and CAAl were −95.83 and −86.22 kJ mol^−1^, respectively, suggesting that boron removal involves strong attractive forces, and the mechanism probably follows a chemical reaction (which can be represented with the reaction of the OH^−^ groups in the cis position). The negative values of ΔS° correspond to a decrease in the degrees of freedom of the sorbed species. The positive values of ΔG° imply that the adsorption of boron on CA and CAAl is not spontaneous; these results are comparable with the results reported in the literature [56].

### 4.6. Sorbent Regeneration

**Figure 7:**
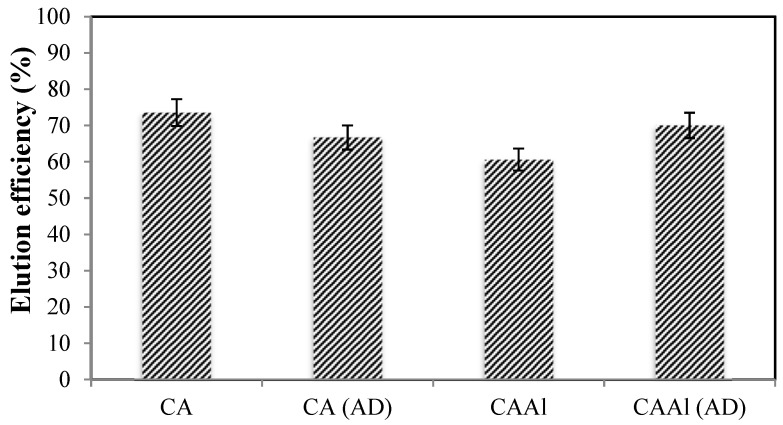
Elution of the sorbents using dilute solution of HCl as eluent. (AD: Air-dried sorbent, eluent: dilute HCl solution (distilled water at pH 3), [B]initial = 50 mg·L^−1^, V = 100 mL).

A sorbent material designed for large-scale applications needs to be reusable in order to be competitive. Figure 7 reports the elution of boron using dilute solution of HCl (distilled water at pH 3) as eluent. The recovery uptake from the loaded sorbents was in the order of 60–75% (the sorption step was carried out at equilibrium pH 9.5, in which the maximum boron removal is performed). Thus, it is possible to use a very cheap eluent to regenerate the CA and CAAl materials; this is a clear advantage in comparison with several commercial sorbents (in water treatment processes) needing expensive solvents to be regenerated (e.g., Dowex-2X8 resin, special effort is required to elute boron from this resin) [57]. A complete desorption study using real effluents (in a continuous system) will be performed in a separate work.

## 5. Conclusions

The effectiveness of CAAl composite material was studied in this work, and its properties as a promising sorbent for boron removal from aqueous solutions were highlighted. The results of CAAl sorbent were compared with those of the individual components (alumina and calcium alginate). The sorption efficiency is highly impacted by the pH of the solutions, which in conjunction with the initial boron concentration and the required contact time, represent the main parameters to take into consideration for up-scaling of the process. The maximum sorption capacities were obtained as 4.5 mmol g^−1^ and 5.2 mmol g^−1^ using CA and CAAl, respectively at equilibrium pH 9.5. The experimental results of the equilibrium data were better fitted with the Langmuir equation (in comparison with the Freundlich model).

Additionally, it was demonstrated that sorption of boron onto CA and CAAl is strongly influenced by the temperature; the exothermic nature of the process was verified (the equilibrium constant decreased 6–7 times with an increase of the temperature from 293 K to 308 K). The type of alginate (with different composition in M and G-blocks) did not influence the boron sorption capacity. The kinetic experimental data can be described using the pseudo-second order rate equation (PSORE). It was found that the uncontrolled air-drying of the sorbents may affect the intraparticle diffusion coefficients. Furthermore, the incorporation of alumina particles into the new composite alginate-based enhances the mechanical strength of the resulting sorbent and enhances the boron removal. The regeneration of the sorbents can be efficiently performed with dilute HCl solution (distilled water at pH 3) with a performance in the order of 60–75% of boron recovery from the loaded sorbent.

## Figures and Tables

**Table 1:** Langmuir and Freundlich parameters of the sorbents for boron removal.

Experimental		Langmuir			Freundlich		
Sorbent	*q*_exp_ (mmol g^−1^)	*q*_max_ (mmol g^−1^)	*k*_L_ (L mmol^−1^)	*r* ^2^	*k*_F_ (mmol^1-1/*n*^ g^−1^ L^1/*n*^)	*n*_F_	*r* ^2^
CA	1.88	4.56	1.36 × 10^−2^	0.989	2.83	2.16 × 10^−2^	0.988
CA (Air-dried)	1.29	1.99	2.72 × 10^−2^	0.950	1.41	3.41 × 10^−2^	0.941
CAAl	1.81	5.21	1.07 × 10^−2^	0.995	3.10	1.75 ×1 0^−2^	0.995
CAAl (Air-dried)	1.18	1.48	4.88 × 10^−2^	0.966	1.13	5.37 × 10^−2^	0.949
Alumina	0.41	0.59	4.64 × 10^−2^	0.994	5.02 × 10^−2^	1.82	0.974

**Table 2:** Langmuir and Freundlich parameters of the sorbents for zinc removal.

Experimental		Langmuir			Freundlich		
Sorbent	*q*_exp_ (mmol g^−1^)	*q*_max_ (mmol g^−1^)	*k*_L_ (L mmol^−1^)	*r* ^2^	*k*_F_ (mmol^1-1/*n*^ g^−1^ L^1/*n*^)	*n*_F_	*r* ^2^
Alumina	0.55	0.71	0.57	0.970	0.22	1.99	0.950
CAAl	1.15	1.33	1.06	0.975	0.58	2.33	0.991

**Table 3:** Comparison of boron sorption capacities for several sorbents.

Sorbents	Temperature (K)	pH	q_max_ (mmol g^−1^)	References
Glycidol-magnesium ferrite (GMF)	298	7.0	6.4	Oladipo and Gazi [44]
Calcined magnesite tailing	318	6.0	6.1	Kipçak and Özdemir [45]
NanoFe	298	8.3	6.01	Zelmanov and Semiat [46]
ChiNi(II)	298	7.0	5.68	Demey et al. [41]
CAAl	298	9.5	5.21	This work
Polyethylenimine-epichlorohydrin resin	298	9.0	5.09	Sarri et al. [47]
Fe-impregnated granular activated carbon (GAC)	298	8.3	4.63	Zelmanov and Semiat [46]
Chitosan/N-Methylglucamine	298	7.0	3.25	Wei et al. [48]
Polymer supported with iminodipropylene glycol functions	298	6.0	3.0	Senkal and Bicak [49]
Zirconium–Chitosan	298	6.0	2.3	Kluczca et al. [50]
Glycosylated polysulfone membrane (GlyPSF)	303	7.0	2.09	Meng et al. [51]
Silica-supported N-methyl-D-glucamine (Si-MG)	298	7.0	1.54	Xu et al. [52]
Ceria–Chitosan	298	7.0	1.3	Kluczca et al. [53]
Alginate/PEI/tannic acid material	298	5.0	0.89	Bertagnolli et al. [54]
Amberlite IRA-743	298	7.0	0.71	Wei et al. [48]
Fly ash	318	10.0	0.64	Polowczyk et al. [55]
Calcined Alunite	298	10.0	0.31	Kavak [19]

**Table 4:** Kinetic parameters of CA and CAAl materials.

Experimental		Pseudo-First Order Model (PFORE)			Pseudo-Second Order Model (PSORE)			Weber & Morris equation
Sorbent	*q*_exp_ (mmol g^−1^)	*k*_1_ (min^−1^)	*q*_1_ (mmol g^−1^)	*r* ^2^	*k*_2_ (g mmol^−1^ min^−1^)	*q*_2_ (mmol g^−1^)	*r* ^2^	*Kp* (mmol g^−1^ min^−1/2^)
CA	0.22	0.23	0.21	0.941	1.66	0.22	0.989	0.29
CA (Air-dried)	0.17	4.54 × 10^−3^	0.16	0.998	2.72 × 10^−2^	0.19	0.999	8.52 × 10^−2^
CAAl	0.18	0.19	0.18	0.932	1.62	0.18	0.986	0.25
CAAl (Air-dried)	0.19	1.15 × 10^−2^	0.17	0.955	7.84 × 10^−2^	0.19	0.988	9.56 × 10^−2^

**Table 5:** Thermodynamic parameters of the sorbents.

Sorbent	Temperature (K)	Kc	$\Delta G{}^{\circ}\;\left(\frac{\mathrm{kJ}}{\mathrm{mol}}\right)$	$\Delta H{}^{\circ}\;\left(\frac{\mathrm{kJ}}{\mathrm{mol}}\right)$	$\Delta S{}^{\circ}\;\left(\frac{\mathrm{kJ}}{\mathrm{mol}}\right)$
CA	293	6.80 × 10^−2^	6.54	−95.82	−0.34
	308	1.01 × 10^−2^	11.78		
CAAl	293	6.03 × 10^−2^	6.84	−86.22	−0.31
	308	1.08 × 10^−2^	11.60		

## Supplementary Materials

The following are available online at https://www.mdpi.com/2073-4360/11/9/1509/s1. Figure S1. Scheme of the manufacturing process of CA and CAAl sorbents. Figure S2. Thermogravimetric analyses. S2a. TGA curve of CA. S2b TGA curve of CAAl. Figure S3. Species of boron and aluminum as a function of pH. Figure S4. Boron sorption isotherms using three different alginates and commercial resin AMBERLITE-IRA 743 (pH 9.5, V = 100 mL, T room, 1 atm). Figure S5. Intraparticle diffusion plot for boron removal. Figure S6. SEM Images of the air-dried sorbents. S6a. Calcium alginate (CA) beads. S6b Composite alginate-alumina (CAAl) beads. Table S1. Main features of AMBERLITE IRA-743.

## Abbreviations

CA	calcium alginate beads
CAAl	composite alginate–alumina
