# Supplementary material for: Boron Removal from Aqueous Solutions by Using a Novel Alginate-Based Sorbent: Comparison with Al2O3 Particles
Source: Polymers (Basel). 2019 Sep 16;11(9):1509. doi: 10.3390/polym11091509 (PMC6780931; doi:10.3390/polym11091509)
Supplement: Supplementary file 1 [file polymers-11-01509-s001.zip › si/polymers-580518-9.16 si.docx]

**SUPPLEMENTARY MATERIALS**


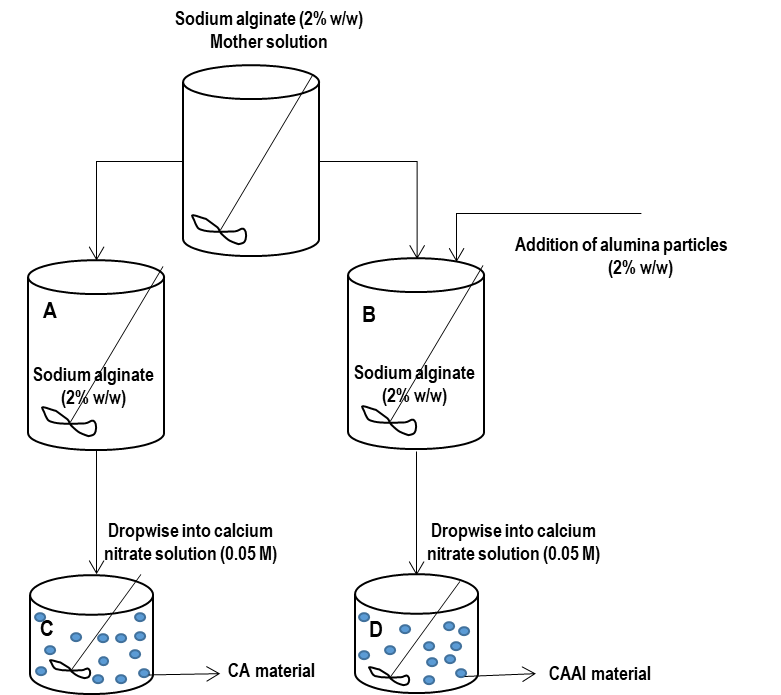


**Figure S1. Scheme of the manufacturing process of CA and CAAl sorbents**


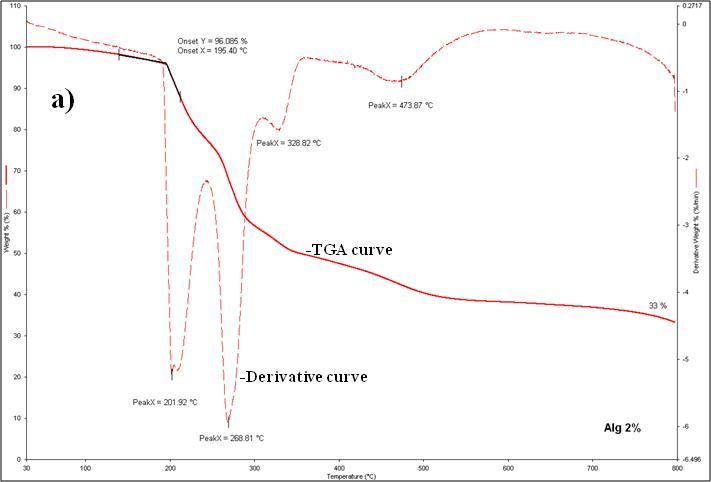


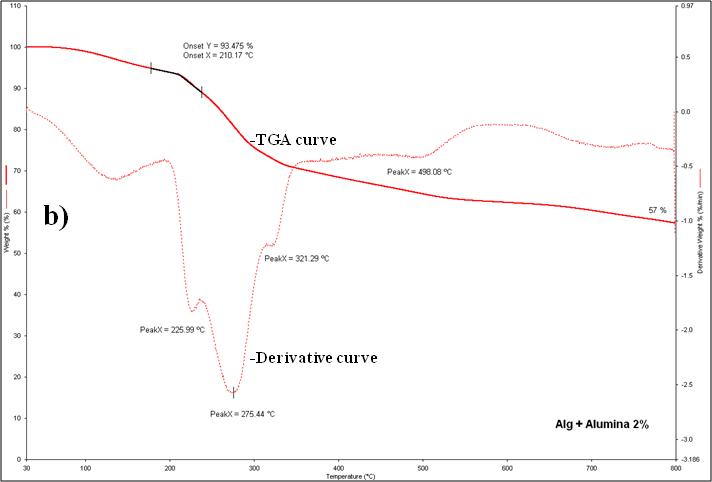


**Figure S2. Thermogravimetric analyses. S2.a. TGA curve of CA. S2.b TGA curve of CAAl**

| 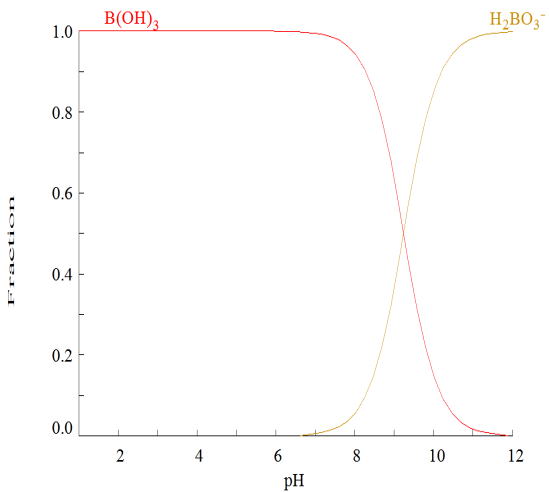 |  |
| --- | --- |

**Figure S3. Species of boron and aluminum as a function of pH**


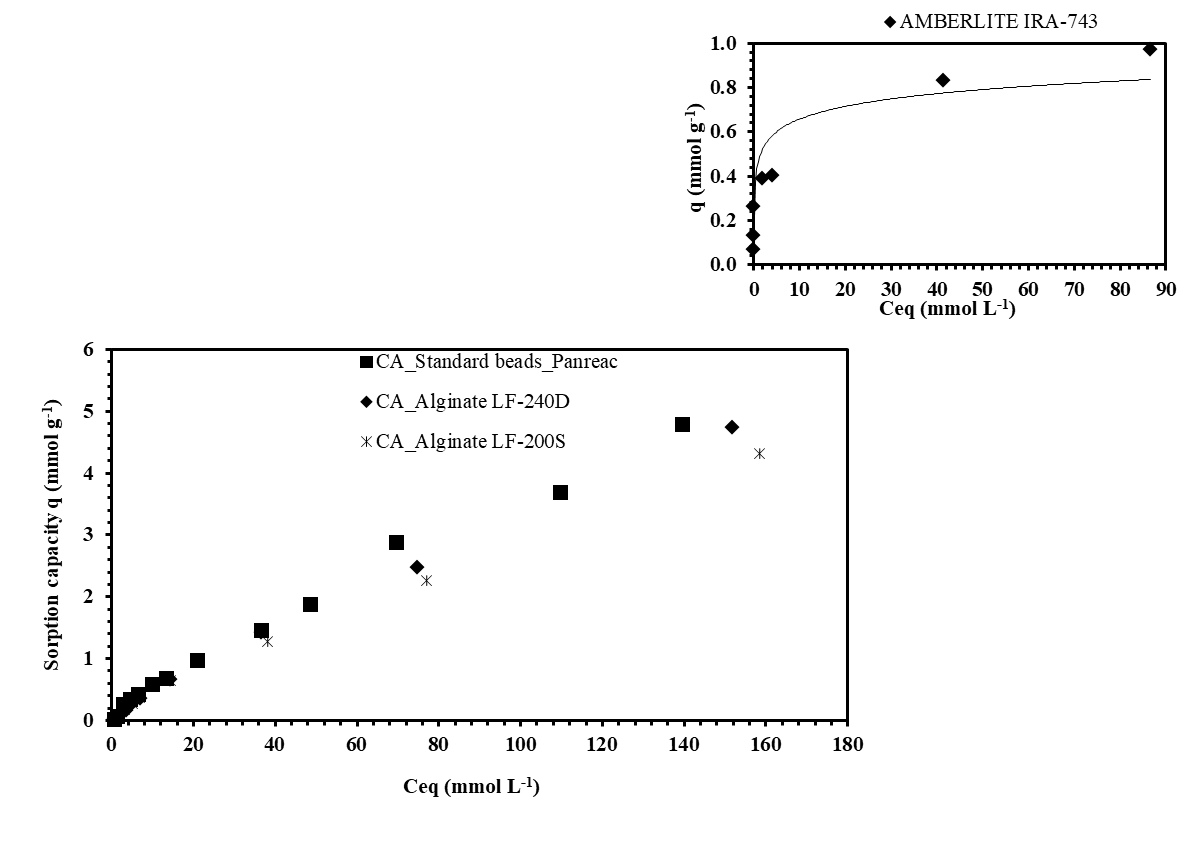


**Figure S4. Boron sorption isotherms using three different alginates and commercial resin AMBERLITE-IRA 743** (pH 9.5, V = 100 mL, Troom, 1 atm).

**Figure S5. Intraparticle diffusion plot for boron removal**


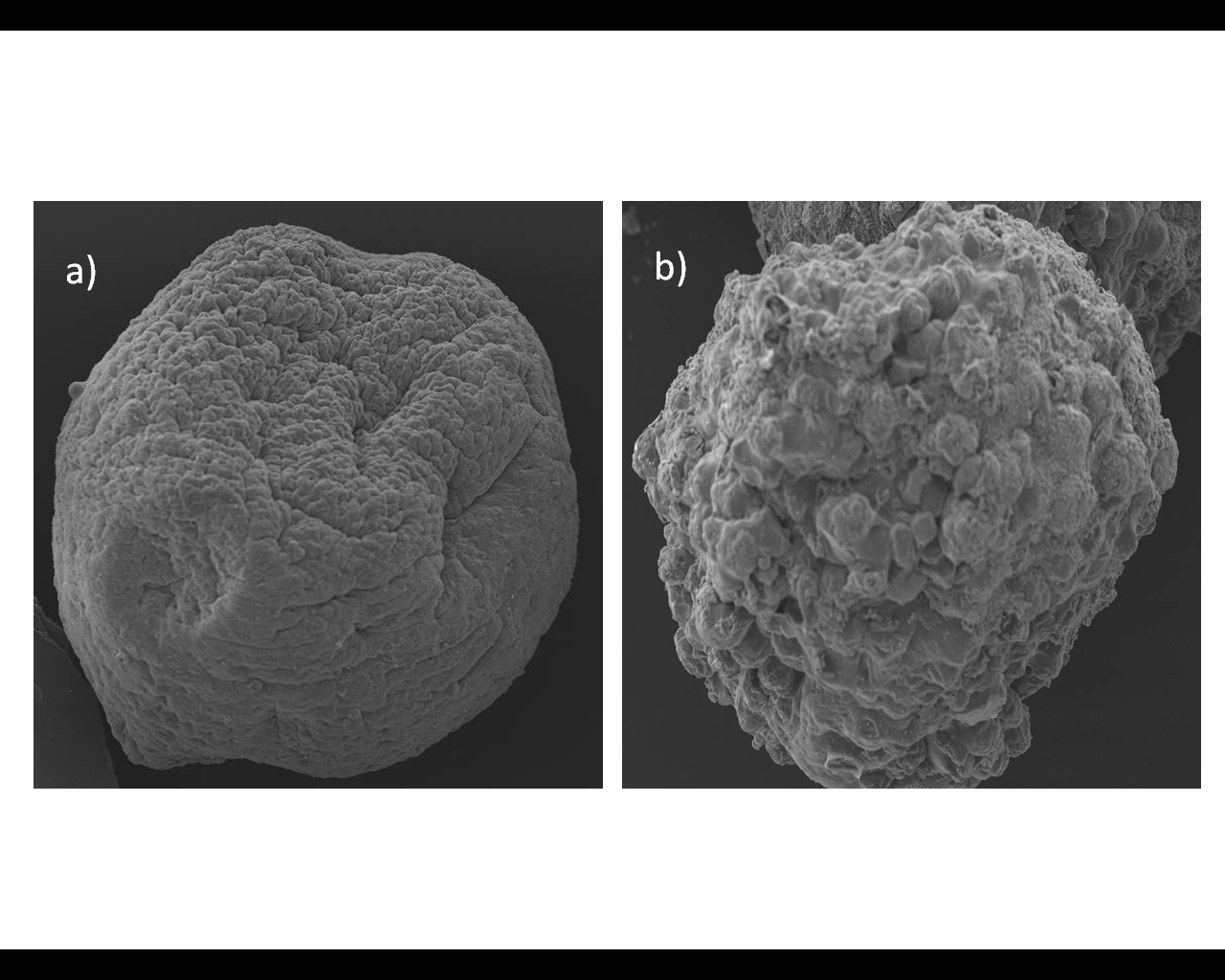


**Figure S6. SEM Images of the air-dried sorbents. S6.a. Calcium alginate (CA) beads. S6.b Composite alginate-alumina (CAAl) beads**

**Table S1. Main features of AMBERLITE IRA-743**

| **Properties** | |
| --- | --- |
| Matrix | Macroporous polystyrene |
| Functional group | N-Methylglucamine |
| Ionic form | Free base (FB) |
| Total capacity | 0.7 eq/L |
| Particle size | 0.5-0.7 mm |
| Moisture holding capacity | 48 to 54 % (FB form) |
